# The dementia fear among older adults: a scoping review

**DOI:** 10.3389/fmed.2026.1938388

**Published:** 2026-10-12

**Authors:** Lin Wang, Guihua Xiong, Xue Dong

**Affiliations:** 1 Changchun University of Chinese Medicine, Changchun, China; 2 College of Rehabilitation Medicine, The Third Affiliated Clinical Hospital of Changchun University of Chinese Medicine, Changchun, China

**Keywords:** assessment tools, dementia fear, dementia worry, influencing factors, intervention, older adults, scoping review

## Abstract

**Background:**

Previous studies evaluating dementia fear among older adults showed inconsistent results regarding its prevalence, measurement approaches, and associated factors. We performed a scoping review to systematically map the literature on measurement tools, prevalence levels, associated factors, and intervention strategies.

**Methods:**

We systematically searched PubMed, Embase, the Cochrane Library, Web of Science, CNKI, Wanfang, VIP, and SinoMed from inception to May 2026. Two reviewers independently screened literature and extracted data using a standardized form. Evidence was synthesized narratively.

**Results:**

Nineteen studies including over 9,000 participants were included: 11 cross-sectional surveys, 2 RCTs, 2 scale development studies, 1 longitudinal study, 1 experimental study, and 2 qualitative studies. Measurement tools were highly heterogeneous; Eight studies used validated multidimensional scales (FADS, FODS-K, DWAS), two used the validated unidimensional Dementia Worry Scale (DWS), while the remaining nine studies relied on single-item or adapted measures Dementia fear was predominantly mild to moderate, but the distribution of study populations was uneven across severity levels. Female gender and depressive symptoms were the most consistently associated factors, though causal directions remained undetermined. Dementia knowledge was positively associated with dementia fear, suggesting knowledge-based strategies alone may amplify rather than alleviate fear. Intervention evidence was extremely limited; only two small-sample RCTs provided preliminary efficacy data.

**Conclusions:**

Current evidence from existing studies did not support a unified conceptual framework or standardized measurement approach for dementia fear. Future research should develop cross-culturally validated multidimensional instruments, conduct longitudinal studies, and design large-sample intervention trials for different worry severity subgroups.

**Systematic Review Registration:**

https://osf.io/r54jk/, identifier [10.17605/OSF.IO/R54JK]

## 1 Introduction

As the global population ages at an accelerating rate, dementia has become one of the major chronic diseases threatening the health and quality of life of older adults. With the rising prevalence of dementia and increased public awareness, individuals' concerns and fears about developing dementia (i.e., dementia fear) have garnered growing attention in academic circles and are regarded as a widespread psychosocial phenomenon (1–3). Within the field, dementia fear is defined as an individual's emotional response to the perceived threat of developing dementia, encompassing both affective components (such as fear) and cognitive components (such as thoughts, mental imagery, and rumination), with its intensity ranging from transient mild concern to severe preoccupation and even phobia (1). This concept differs from “anticipatory dementia” (2); it is essentially a combination of health anxiety and aging anxiety and can occur in individuals of any age or cognitive status (1). However, the use of terminology regarding dementia fear remains somewhat inconsistent in the academic community; terms such as “fear,” “worry,” “anxiety,” “concern,” and “perceived threat” are often used interchangeably, and there is a lack of a unified operational definition (3), which poses significant obstacles to research integration and cross-cultural comparisons.

Epidemiological studies indicate that dementia fear is quite common among the elderly, but estimates of its prevalence vary widely across existing studies. Surveys in the United States show that 26% to 49% of respondents are concerned about developing Alzheimer's disease (4, 5); surveys across multiple European countries have found that dementia is one of the diseases the public fears most (6); meanwhile, a multicenter survey in China revealed that 76.7% of the general public experiences dementia fear (7). Beyond general population estimates, psychological predictors of participation in cognitive impairment screening have also highlighted the role of fear in older adults' health behaviors (8). The negative consequences of excessive fear are further evidenced by its association with suicidal ideation among older adults (9), and its presence even among memory clinic patients without organic mental disorder diagnoses (10). This wide variation in prevalence estimates and clinical manifestations stems largely from inconsistencies in conceptual definitions and measurement methods, making it difficult to determine the true extent and distribution characteristics of this phenomenon (3).

With regard to assessment tools, early studies often used a single item to measure dementia fear; while convenient, such approaches make it difficult to ensure reliability and validity, and they fail to reflect the multidimensional nature of this construct (3). In recent years, multidimensional assessment tools have gradually emerged. Early research employing the Perceived AD Threat Scale examined the role of personal experience with Alzheimer's disease in shaping perceived threat (11). Subsequently, a review of research progress on dementia fear assessment tools has documented the evolving landscape of these instruments (12). The Perceived AD Threat Scale was first reported in a study of illness representations among first-degree relatives (13). The Fear of Alzheimer's Disease Scale (FADS) was developed to assess anticipatory dementia in older adults (14). The Dementia Worry Scale has been used to examine the relationship between dementia worry, exposure, psychological factors, and subjective memory concerns (15). The Fear of Dementia Scale (FOD; Page et al., 2019) represents a 35-item multidimensional measure examining fear of dementia, with a three-factor structure (burden and loss, quality of life, and perceived social and cognitive loss) (16). Independently, Lee and Jung (2020) developed the Fear of Dementia Scale (FODS-K) for community-dwelling older adults in South Korea; this 19-item instrument comprises a three-dimensional, five-factor structure (cognitive, social, and physical domains) and was validated in a Korean population (17). Despite their similar names, FOD and FODS-K are distinct instruments developed by separate research teams in different cultural contexts. These tools assess the construct from cognitive, emotional, physical, and social dimensions, respectively; however, they vary significantly in the number of items, scoring methods, and target populations. Furthermore, most studies still tend to use total scores while neglecting their multidimensional structure (3). In addition, the cultural appropriateness of existing scales has not been fully validated, limiting their application across different populations (12).

Existing research has revealed the complex factors influencing dementia fear and its adverse consequences. Demographically, women, individuals with lower educational attainment, and those with a family history of dementia or experience caring for someone with dementia report higher levels of fear (1, 18, 19). In terms of health and psychological factors, subjective memory complaints, poor self-rated health, symptoms of depression and anxiety, and anticipated stigma are all significantly associated with dementia fear (15, 20, 21). Cognitive factors such as knowledge about dementia, risk perception, and expectations regarding aging also play an important role (22, 23). However, most existing studies employ cross-sectional designs, making it difficult to determine the direction of causality. In terms of adverse consequences, excessive dementia fear may lead to avoidance of medical care and delayed diagnosis (9), reduce quality of life and exacerbate psychological distress (10), and may even be associated with suicidal ideation (9). Furthermore, high levels of anticipated stigma may intensify dementia fear, which in turn affects social functioning and help-seeking behavior (21).

Although there is preliminary awareness of the negative consequences of dementia fear, research on interventions targeting this phenomenon remains scarce. The existing literature largely focuses on assessment and analysis of associated factors, with a lack of systematic development of intervention strategies and evaluation of their effectiveness (3, 12). A few studies suggest that reducing stigma-related perceptions associated with dementia may help alleviate anxiety levels (21). Therefore, synthesizing existing intervention studies and exploring effective coping strategies has become an urgent and important task in this field (3, 12).

In summary, the concept of dementia fear is poorly defined, measurement tools are complex, estimates of its prevalence vary, the causal relationships between risk factors and adverse outcomes are unclear, and there is a severe lack of evidence on interventions. These issues collectively hinder progress in this field. Given the heterogeneity of study designs, measurement instruments, and outcome definitions in this emerging field, a systematic review with meta-analysis was deemed inappropriate at this stage. Instead, a scoping review was chosen to systematically map the breadth of existing evidence, clarify key concepts, examine heterogeneous literature, identify knowledge gaps, and inform the development of more focused systematic reviews in the future (24, 25). Therefore, this scoping review aims to systematically search for, synthesize, and analyze the existing literature on dementia fear among older adults, focusing on answering the following four questions: (a) What is the current status of dementia fear among older adults? (b) What are its risk factors and adverse consequences? (c) What assessment tools are currently available, and what are their psychometric properties? (d) What intervention methods are currently available, and what are their effects and limitations? By addressing these questions, this study aims to provide an evidence-based foundation for establishing a unified theoretical framework for dementia fear care, developing localized assessment tools, and formulating targeted intervention strategies.

## 2 Methods

This scoping review was conducted following the methodological framework of Arksey and O'Malley (24) and its enhancement by Levac et al. (25), and reported in accordance with the PRISMA Extension for Scoping Reviews (PRISMA-ScR) (Tricco et al., 26). Eight databases were systematically searched because of their relevance to the topic. These included PubMed, Embase, the Cochrane Library, Web of Science, China National Knowledge Infrastructure (CNKI), Wanfang, VIP, and SinoMed. The literature search was limited to Chinese- and English-language, peer-reviewed journal articles published from inception to May 2026. The study protocol was registered on the Open Science Framework (OSF) and is available at https://osf.io/r54jk/ (Project DOI: 10.17605/OSF.IO/R54JK).

### 2.1 Literature search

Searches included the following key terms and Boolean connectors. For English databases (taking PubMed as an example): ((((((dementia fear[MeSH Terms]) OR (fear of dementia[Title/Abstract])) OR (dementia worry[Title/Abstract])) OR (fear of Alzheimer's disease[Title/Abstract])) OR (fear of Alzheimer[Title/Abstract])) OR (dementia phobia[Title/Abstract])) AND (((Aged[Title/Abstract]) OR (elderly[Title/Abstract])) OR (older[Title/Abstract])). For Chinese databases (taking CNKI as an example): (SU=“old age”+‘elderly’) AND (SU=“dementia fear”+“concerns about dementia”+“anxiety about dementia”+“anticipatory dementia”). The English and Chinese search strings were developed independently by bilingual researchers to optimize sensitivity in each linguistic and indexing context, rather than being direct translations of one another. Specifically, “dementia phobia” was omitted from the Chinese string because no established equivalent term is indexed in Chinese databases; conversely, “anticipatory dementia” was retained in the Chinese string as it represents a recognized construct in Chinese-language gerontological literature. The search was restricted to English- and Chinese-language publications because the review team was proficient only in these two languages. Records retrieved from the electronic searches were imported into EndNote for deduplication and screening. The electronic searches were augmented by hand searching reference lists of included studies to ensure a thorough search.

### 2.2 Selection process

The following inclusion criteria were used for article selection: a. participants aged 60 years and older; b. addressed dementia fear, dementia worry, or related constructs in older adults; c. involved assessment, correlates, or intervention strategies for dementia fear; d. published in Chinese or English; and e. original empirical studies. We excluded duplicate publications, articles for which full text could not be retrieved, reviews, systematic reviews, conference abstracts, policies, and guidelines.

Identified records were screened independently by two researchers using EndNote for deduplication and title/abstract screening, with cross-checking between the two. Disagreements were resolved through discussion or by consulting a third researcher when necessary. Both title/abstract screening and full-text eligibility assessment were performed independently by two reviewers (LW and GX). Disagreements were resolved through discussion or by consulting a third researcher (XD) when necessary.

### 2.3 Data extraction

Data for each study included in the review were extracted to a standardized Excel table, including general characteristics of each article (authors' names, year of publication, study location, the aim of the study, study design, characteristics of the participants, measurement tools, and a summary of the main results), together with details regarding the study theme and key outcomes. A standardized data extraction form was developed *a priori* by the research team based on the review questions and pilot-tested on three randomly selected included studies. Two reviewers (LW and GX) independently extracted data from all included studies. Any discrepancies were resolved through discussion or by consulting a third reviewer (XD) when consensus could not be reached.

### 2.4 Data analysis

Before determining the way articles would be presented and analyzed, two researchers independently extracted the data, and all authors reviewed and agreed upon the contents included in the table. Information including the measurement of dementia fear, its prevalence level and distribution, associated factors, and intervention effects were synthesized narratively. Given the scoping review aim to map existing evidence and identify research gaps, no formal quality assessment or risk-of-bias appraisal was conducted. Narrative synthesis was conducted by grouping studies according to study design (cross-sectional, longitudinal, experimental, qualitative, scale development, and RCT) and thematic domain (measurement, prevalence, associated factors, and interventions). Themes and categories were derived inductively from the extracted data through iterative discussion among all authors. Summary tables were created to facilitate comparison across studies. Consistent with scoping review methodology, no formal risk-of-bias or methodological quality assessment was conducted, as the primary aim was to map the available evidence and identify research gaps rather than to evaluate study quality or synthesize effect estimates (24).

## 3 Results

A total of 19 studies examining dementia fear among older adults met the inclusion criteria. Most studies employed a cross-sectional design (*n* = 11); two were randomized controlled trials (RCTs) (29, 37); two were scale development and validation studies (14, 17); one was a longitudinal secondary data analysis (28); one was an experimental study (38); and two were qualitative studies (27, 40). Most studies were conducted in the United States (*n* = 5), China (*n* = 5), and Germany (*n* = 2), with the remainder distributed across the United Kingdom, South Korea, Japan, Egypt, Belgium, and Israel. Sample sizes ranged from 15 participants (qualitative studies) to approximately 3,890 participants (nationwide representative surveys), with a total sample size exceeding 9,000. The literature screening process is shown in Figure 1.

**Figure 1:**
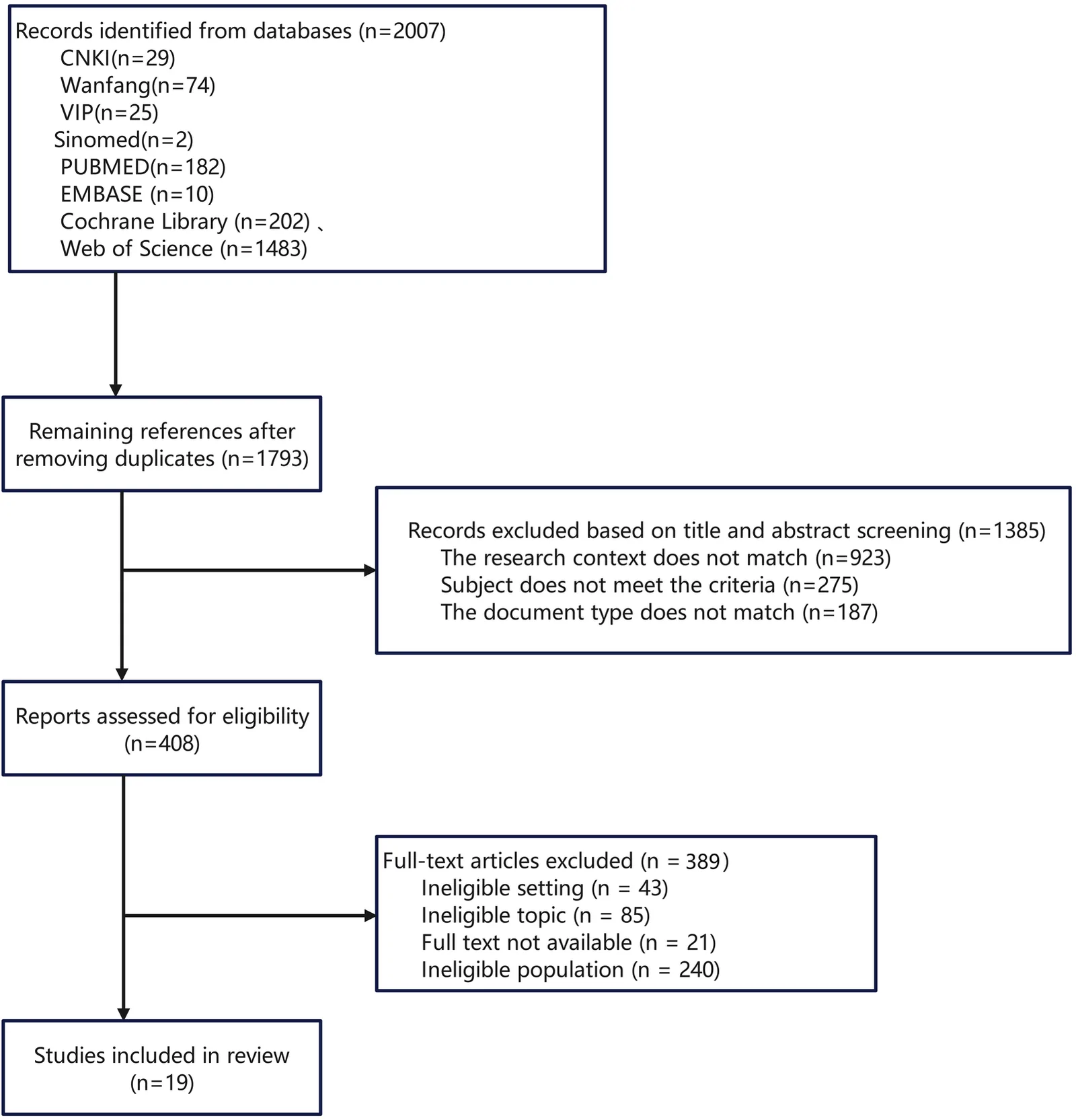
PRISMA-ScR flow diagram of the literature screening and selection process. A systematic search of eight electronic databases (CNKI, Wanfang, VIP, SinoMed, PubMed, EMBASE, Cochrane Library, and Web of Science) yielded 2,007 records. After removing 214 duplicates, 1,793 records remained for title and abstract screening, of which 1,385 were excluded (923 for ineligible research context, 275 for ineligible participants, and 187 for ineligible document type). The remaining 408 reports were assessed for eligibility in full text. Of these, 389 full-text articles were excluded (43 for ineligible setting, 85 for ineligible topic, 21 for unavailable full text, and 240 for ineligible population). Ultimately, 19 studies were included in the scoping review.

### 3.1 Basic characteristics of the included studies

#### 3.1.1 Research objectives and topics

Most studies (*n* = 13) aimed to assess levels of dementia fear and explore its associated factors. Specifically, 9 studies focused on demographic, psychosocial, and health-related factors influencing dementia fear (20, 27–36, 39); 1 study used a longitudinal design to examine the predictors of dementia fear and its trajectories (28); two studies examined the association between dementia fear and cognitive function or neuropsychological performance (38, 41); and one study investigated the chain mediation mechanism between dementia fear and insomnia (42). In addition, two studies were intervention-focused, evaluating the efficacy of psychoeducation or cognitive behavioral therapy in reducing dementia fear (29, 37). Two qualitative studies explored older adults' dementia fear and their cognitive experiences from the perspective of lived experience (27, 40). Only two studies were primarily aimed at developing scales (14, 17). Specific characteristics of the literature are presented in Table 1.

**Table 1:** Basic characteristics of the included literature.

Studies	Year	Country	Study design	Population	Sample size (n)	Assessment indicators	Study aim	Key findings
French (14)	2012	USA	Scale development/validation	Community-dwelling older adults	101	①③	Develop and validate a fear scale for assessing anticipatory dementia in older adults	Developed FADS scale, 30-item three-factor structure (General Fear, Physical Symptoms, Catastrophic Attitude), Cronbach's *α* = 0.94
Lee (M. Lee) & Jung (17)	2020	South Korea	Scale development/validation	Community-dwelling older adults	250	①③	Develop and validate a fear of dementia scale for community-dwelling older adults	Developed FODS-K scale, 19-item three-dimension five-factor structure, explaining 78.44% of total variance
Bowen (20)	2019	Germany	Cross-sectional study	Older adults	219	①③⑥	Explore the relationship between dementia worry and sociodemographic, health-related, and psychological factors	41.1% reported dementia worry; significantly associated with psychological distress, perceived memory change, aging anxiety, and personal risk perception; age showed an inverted U-shaped relationship with worry
Corner & Bond (27)	2004	UK	Qualitative study	Older adults	15	①	Explore fears and anxieties of older adults regarding dementia risk	Respondents generally associated memory decline with dementia; main concerns included loss of self, dignity, independence, and institutionalization
Cutler & Bragaru (28)	2015	USA	Longitudinal study	Older adults	1,725	①②⑥⑦	Explore long-term and short-term predictors of worries about developing Alzheimer's disease	Short-term predictors: depression, age, genetic beliefs; long-term poorer self-rated cognitive function predicted higher AD worry
Farina et al. (29)	2023	USA	RCT	Community-dwelling older adults	81	①③⑥	Evaluate the effect of a brief psychological intervention (REFRAME) on promoting adaptive coping with ADRD-related fear and avoidance	Both groups showed significant reductions in FAM and FADS scores, but no significant between-group difference; REFRAME group showed greater depression reduction
Joo et al. (30)	2021	South Korea	Cross-sectional study	Community-dwelling older adults	1,869	①⑥⑦	Explore dementia knowledge, dementia worry, and related factors	84.8% had dementia worry; dementia knowledge positively correlated with worry; writing/music cognitive activities associated with lower worry
Bardach (31)	2021	USA	Cross-sectional study	Community-dwelling older adults	502	①⑥	Explore the relationship between dementia worry, fear, stigma, and engagement in genetic and interventional research	Higher AD worry and lower stigma fear associated with current AD observational study volunteer status; AD worry not associated with clinical trial participation
Han et al. (32)	2023	China	Cross-sectional study	Nursing home older adults	550	①③④⑥	Explore the current status and influencing factors of dementia fear among nursing home older adults	FODS-K score 56.50 ± 10.73, moderately high; negatively correlated with health-promoting lifestyle, positively correlated with loneliness; influencing factors explained 60.8% of variance
Bai et al. (33)	2024	China (Shaanxi)	Cross-sectional study	Hospitalized older adults	458	①③④	Explore the current status and influencing factors of dementia fear among hospitalized older adults	FODS-K median 54.00; chronic disease types, monthly income, presence of dementia patients nearby, whether current disease may cause dementia, self-rated health, and depression were influencing factors
Meyer et al. (34)	2024	Germany	Cross-sectional study	Older adults	3,890	①③⑤⑥	Explore the frequency and related factors of dementia fear	12.7% severe fear, 34.6% mild; higher fear significantly associated with female gender, older age, not living with partner, depression, and anxiety symptoms
Khalil et al. (35)	2025	Egypt	Cross-sectional study	Community-dwelling older adults	1,067	①⑤⑥⑦	Explore the influence of dementia knowledge and personal dementia fear on risk reduction and screening participation	Dementia fear mean 27.16 ± 8.91; positively correlated with lifestyle change motivation and memory screening acceptance; high knowledge combined with high fear may weaken behavioral intention
Guo et al. (36)	2025	China	Cross-sectional study	Hospitalized older adults	263	①③④	Explore the current status and latent profiles of dementia fear among hospitalized older adults	Identified 3 latent classes: mild 52.9%, fluctuating moderate 21.3%, cognitively sensitive-severe 25.8%
An (37)	2020	China/Hong Kong	RCT	Nursing home older adults	82	①③	Evaluate the effectiveness of modified group CBT on dementia worry among Chinese older adults	Modified group CBT significantly reduced dementia worry (g = −1.49) and cultural bias beliefs (g = −0.95); those with less than primary education benefited most
Fresson et al. (38)	2017	Belgium	Experimental study	Community-dwelling older adults	72	①②	Explore the moderating effect of dementia worry on cognitive performance under stereotype threat	Stereotype threat reduced executive function performance; high dementia worry participants showed worse executive function under threat, low worry participants showed no significant effect
Nakayama et al. (39)	2025	Japan	Cross-sectional study	Community-dwelling older adults	299	①③⑥⑦	Explore influencing factors of dementia worry among community-dwelling adults in Japan	Dementia knowledge, number of somatic symptoms, depression severity, and parental dementia diagnosis were significant predictors; model explained 16.4% of variance
Lund et al. (40)	2025	UK	Qualitative study	Older adults	35	①③	Explore differences in metacognitive beliefs between older adults with high and low dementia worry	High worry group focused on self-consequences and fear; low worry group focused on others and coping; essential differences in metacognitive beliefs between groups
Caughie et al. (41)	2021	USA	Cross-sectional study	Community-dwelling older adults	40	①②	Explore the relationship between dementia worry and neuropsychological performance in healthy older adults	High worry participants showed worse executive function performance; young-old (65-75) had significantly higher dementia worry than old-old (76–85)
Dai et al. (42)	2023	China	Cross-sectional study	Community-dwelling older adults	259	①③④⑥	Explore the relationship between dementia fear and insomnia, and the mediating effects of social isolation and resilience	FODS-K mean 55.32 ± 9.42; fear positively correlated with insomnia; social isolation and resilience played chained mediating roles between fear and insomnia

① Dementia fear-related indicators; ② Cognitive function-related indicators; ③ Psychological and mental status; ④ Social support factors; ⑤ Health behaviors and motivation; ⑥ Health-related factors; ⑦ Dementia knowledge.

FODS-K = Fear of Dementia Scale–Korean version (Lee & Jung, 17); distinct from FOD (Page et al., 16).

#### 3.1.2 Research design and data collection methods

Cross-sectional questionnaire studies were predominant (*n* = 11), of which 4 studies used convenience sampling (20, 27, 33, 42), 3 studies used multistage or quota sampling to enhance representativeness (34–36), 1 RCT used randomized assignment (29), and 1 cross-sectional study used random-digit-dialing sampling (32). Data collection primarily involved face-to-face interviews and self-administered questionnaires, with some studies using online or mail-in methods (29, 34). Both RCTs included an intervention group and a control group, employing a brief psychological intervention (REFRAME) with remote delivery and a modified group cognitive behavioral therapy (CBT), respectively (29, 37). It is worth noting that all studies had methodological limitations: the cross-sectional design could not establish causality, and the small sample sizes of the RCTs (*N* = 40–82) may have compromised statistical power.

#### 3.1.3 Characteristics of study participants

The study participants were primarily community-dwelling older adults, ranging in age from 55 to 99 years. Most studies used an age of ≥60 or ≥65 years as an inclusion criterion, but some studies included a broader range of middle-aged and older adults; for example, one study included participants aged 50–99 years (28), and another included those aged 40–70 years (34). Female participants constituted the majority in most studies (51%–82.9%). Educational levels varied significantly across studies, ranging from below elementary school to graduate school; some studies included participants with higher levels of education (41). Two studies specifically excluded individuals with cognitive impairment or major depression (29, 41) to ensure that the study participants were cognitively normal but highly anxious about dementia—the “worried well” (health anxiety) group.

### 3.2 Measuring dementia fear

Of the 19 studies, eight used validated multidimensional scales (FADS, FODS-K, DWAS); two used the validated unidimensional Dementia Worry Scale (DWS); and the remaining nine employed single-item measures or self-adapted instruments. The Dementia Worry Scale (DWS) is an eight-item unidimensional instrument assessing the severity of dementia-related worry (37, 41) and is distinct from the 16-item two-factor Dementia Worry Assessment Scale (DWAS) (39). Specifically, the Fear of Alzheimer's Disease Scale (FADS) consists of 30 items organized into a three-factor structure (General Fear, Physical Symptoms, Catastrophic Attitude), with a Cronbach's *α* of 0.94 and factor loadings ranging from 0.40 to 0.86 (14). The Fear of Dementia Scale (FODS-K) consists of 19 items with a three-dimensional, five-factor structure, explaining 78.44% of the total variance (17). The Dementia Worry Assessment Scale (DWAS) consists of 16 items with a two-factor structure, and the total scale *α* is 0.94 (39). The Dementia Worry Scale (DWS) has been adopted in multiple studies (37, 41). Reliability estimates for the DWS were not reported in the included studies. However, most studies (*n* = 12) used single-item measures or self-adapted instruments, such as “Are you worried about developing dementia?” or single-item assessments based on a Likert scale (28, 30). This lack of measurement consistency has severely hindered cross-study comparisons of the true levels of dementia fear. Furthermore, although some studies used multidimensional scales, they reported only total scores in their data analysis (32, 33).

### 3.3 Levels of dementia fear

The levels of dementia fear reported in various studies vary depending on the measurement tools, study populations, and cultural contexts, making direct comparisons difficult. When using single-item measures, reported rates range from 16% to 84.8% (30). When multidimensional scales were used, the mean FADS total score was M = 29.28 ± 18.40 (possible range 0–120) (14), the mean FODS-K total score was M = 55.32 ± 9.42 (range 19–95) (17, 42), and the mean DWAS total score was M = 22.7 ± 13.7 (range 0–64) (39). A nationally representative survey in Germany showed that only 12.7% reported severe dementia fear, while 34.6% reported mild dementia fear (34). Intervention studies reported higher baseline levels, with a baseline FADS mean of M = 46.3 ± 20.3 (range 0–120) (29). Regarding the DWS, the included studies used different versions: An et al. (37) used a 9-item adapted version (range 9–45) with a score of ≥22 as an eligibility criterion, whereas Caughie et al. (41) used the 12-item Kinzer and Suhr version (range 12–60). No established clinical cutoffs for severity classification were reported in the included studies for any of these scales. Qualitative studies further reveal that dementia fear is not a unidimensional form of anxiety, but rather involves deep-seated concerns about the loss of self, dignity, independence, and the burden on one's family (27, 40). There were significant differences in metacognitive beliefs between the high-concern and low-concern groups (M = 36.08 ± 4.94 vs. M = 18.14 ± 4.54) (40).

### 3.4 Factors associated with dementia fear

#### 3.4.1 Demographic factors

Evidence regarding the relationship between age and dementia fear is inconsistent. Most studies report a negative correlation between age and dementia fear, meaning that older adults report lower levels of dementia fear (28, 41). Other studies, however, have found an inverted U-shaped relationship between age and dementia fear, with the peak occurring around age 70 (20, 34). Furthermore, a multiple regression analysis showed that age did not reach statistical significance (*β* = −0.123, *P* = 0.089) (39). These conflicting results may stem from differences in how various studies tested for age effects, as well as variations in measurement tools and sample characteristics.

The evidence regarding gender is relatively consistent. Most studies show that women report significantly higher levels of dementia fear than men (30, 34, 38), with one experimental study (38) providing statistical evidence (*P* = 0.001). However, other studies (39) have found that gender does not significantly predict dementia fear (*β* = 0.103, *P* = 0.087).

Findings regarding educational level are similarly inconsistent. Some studies show that lower educational levels are associated with higher levels of dementia fear, while others find that educational factors are only occasionally significant or not significant at all (34, 39).

#### 3.4.2 Health and psychological factors

Associations between depression, psychological distress, and dementia fear have shown the most consistent evidence across studies. Dementia fear is significantly associated with psychological distress (20) and positively correlated with insomnia (*r* = 0.474, *P* < 0.01) (42). Regression analysis showed that the severity of depression is a significant predictor of dementia fear (*β* = 0.162, *P* = 0.004) (39), and other studies have also reported depression as a factor influencing dementia fear (32, 33). However, since most studies employed cross-sectional designs, the direction of the causal relationship between psychological distress and dementia fear cannot be determined.

The association between subjective health status and dementia fear is also fairly consistent. Most studies show that poorer self-rated health is associated with higher levels of dementia fear (20, 33, 34). Regarding physical symptoms, the number of physical symptoms experienced in the past year was a significant predictor of dementia fear (*β* = 0.161, *P* = 0.007) (39), which is consistent with the findings of other studies (30, 32).

#### 3.4.3 Cognitive and knowledge factors

The relationship between knowledge of dementia and dementia fear is contradictory. Some studies have found a positive correlation between knowledge of dementia and dementia fear (30, 39), and regression analysis further confirmed that the higher the level of knowledge about dementia, the more severe the dementia fear (*β* = 0.155, *P* = 0.006) (39).

The association between subjective memory complaints and dementia fear is also fairly consistent. Longitudinal studies show that consistently poor self-reported cognitive function over time predicts higher dementia fear (28), and previous research has also found a significant association between subjective memory complaints and dementia fear (20). However, the relationship between objective cognitive function and dementia fear remains unclear. Some studies have found that dementia fear primarily affects executive function rather than memory, and is significant only under experimental conditions involving stereotype threat (38, 41).

#### 3.4.4 Social environment and exposure factors

A consistent positive association between a history of exposure to dementia (e.g., a relative with dementia) and dementia fear has been observed across various studies. Multiple studies have reported that individuals with a family history of dementia or personal exposure to it exhibit higher levels of dementia fear (27, 32, 39); one regression analysis showed that a parent's dementia diagnosis was a significant predictor (*β* = 0.117, *P* = 0.034) (39). Regarding social support, social support is negatively correlated with dementia fear, whereas loneliness is positively correlated with dementia fear (32, 36).

#### 3.4.5 Effectiveness of the intervention

Two RCTs provided evidence on interventions to reduce dementia fear. Modified group CBT significantly reduced concerns about dementia (*g* = −1.49, *P* < 0.001) and culturally biased beliefs (*g* = −0.95, *P* < 0.001), both with large effect sizes (37). The REFRAME intervention also showed significant reductions in both FAM and FADS scores across both groups, but the differences between groups were not statistically significant (29). Notably, the effectiveness of the intervention was moderated by educational level: participants with an education level below elementary school benefited the most (*b* = −12.38, *P* < 0.001), whereas the intervention had no significant effect for those with a high school education or higher (*P* = 0.32).

## 4 Discussion

### 4.1 Measurement tools and cross-cultural applicability

When interpreting the results of this review, it is important to recognize that the included studies measured constructs that partially overlapped but were distinct from one another (e.g., fear of dementia, worry about dementia, perceived threat), and that the apparent differences between studies may reflect both scale bias and differences in the actual populations. This scoping review included 19 studies examining dementia fear among older adults. The most fundamental limitation of this field is the heterogeneity of measurement instruments. Only 7 of the 19 studies used validated multidimensional scales; the remainder relied on single-item or self-adapted tools. The three main multidimensional instruments cover markedly different conceptual domains: the FADS (14) focuses on anticipatory anxiety related to Alzheimer's disease; the FODS-K (17) emphasizes cognitive, social, and physical responses; and the DWAS (39) centers on perceived threat and functional impact. These differing foci make direct score comparison difficult, and it remains unclear whether observed differences reflect genuine population variation or instrument bias.

Cross-cultural applicability is also limited. The FODS-K and DWAS were validated only in South Korea and Japan, respectively (17, 39), and evidence for the FADS in non-Western cultures is lacking (14). Conclusions about East–West differences may therefore reflect cultural instrument bias rather than true population differences. Single-item or non-standardized measures are particularly problematic, as the remaining studies using them (20, 28, 30, 31, 34, 35) lose substantial information and cannot distinguish mild worry from severe fear or their differing psychological mechanisms and intervention needs. Furthermore, most studies using multidimensional scales reported only total scores, obscuring the multidimensional structure. Identical totals may mask very different dimensional profiles, and the associated factors and intervention strategies should also differ (14, 17, 39). Existing research has rarely examined this level of heterogeneity.

Cultural context may shape dementia fear through multiple pathways (43). In East Asian cultures, where Confucian values emphasize family filial piety and collective identity, dementia may be perceived not only as a personal loss of self but also as a profound burden on family honor and intergenerational obligations (44). This may intensify fear relative to Western individualistic contexts, where autonomy-based concerns (e.g., loss of independence) may predominate (2). Additionally, differences in dementia stigma, healthcare access, and public awareness campaigns across countries may moderate both the experience and expression of fear (45). Because all included studies were single-country designs, direct cross-cultural comparisons remain impossible, and observed East–West differences should be interpreted cautiously (46).

### 4.2 Levels of dementia fear and population coverage

It should be noted that direct comparison of prevalence estimates across studies is complicated by the heterogeneity of measurement constructs and instruments used. Evidence suggests dementia fear in the general elderly population is predominantly mild to moderate, with severe fear being uncommon (34). However, coverage is imbalanced: general surveys typically report low concern (34), while intervention studies select participants with high concern (29, 37), leaving the moderate group least studied. This imbalance may lead to misallocation of intervention resources. Qualitative studies indicate that dementia fear involves concerns regarding loss of self, dignity, independence, and family burden (27, 40). Individuals with high and low anxiety differ in metacognitive beliefs (40), suggesting the core of this fear may be catastrophic interpretation of cognitive signals. Interventions should therefore focus on altering these interpretation patterns.

### 4.3 Gender and age

These associations should be interpreted cautiously, as the underlying construct measured (fear, worry, or perceived threat) and the cultural context may moderate the observed relationships. Gender is the most consistent cross-cultural demographic predictor. Most studies report higher dementia fear in women than men (30, 34, 38), yet a Japanese study found no significant gender effect (39), suggesting the association may be moderated by sociocultural norms. All included studies are single-country designs, precluding direct cross-cultural comparison.

Age relates to fear inconsistently. Most cross-sectional studies report a negative correlation (28, 41), whereas a German study found an inverted U-shaped pattern peaking around age 70 (20, 34). This discrepancy likely stems from methodological differences; most studies test only linear effects and overlook nonlinearity, while cross-sectional designs confound age with generational effects. Longitudinal validation is needed, but only one such study exists (28), leaving causal inference unsupported.

Several mechanisms may explain the consistently higher dementia fear observed among women. First, women often assume greater informal caregiving responsibilities for relatives with dementia (47), which may increase exposure to disease-related distress and heighten perceived vulnerability. Second, women tend to report higher levels of health anxiety and somatic awareness (48), which may amplify fear of cognitive decline. Third, gendered social norms may make it more acceptable for women to express fear, whereas men may underreport emotional concerns (49, 50). These hypotheses require direct empirical testing in future research.

### 4.4 Psychological, cognitive, and knowledge factors

Given the conceptual heterogeneity of the instruments used across studies, the direction and strength of these associations may depend on which specific dimension of dementia fear was assessed. Depression shows the most consistent association with dementia fear (20, 32, 33, 39), yet cross-sectional designs preclude causal inference. Depression may amplify concerns about cognitive decline through negative cognitive bias, while chronic stress from sustained fear may worsen depression; catastrophizing interpretations of memory lapses may also directly trigger fear. This bidirectional pathway remains unresolved. A chained mediation linking fear to insomnia via social isolation and resilience has been proposed (42), but mediation analysis cannot establish causality, and single-target interventions may have limited impact. Somatic symptoms are also consistently associated with fear (30, 32, 39), though studies have not distinguished the symptoms themselves from patients' interpretations.

Knowledge correlates positively with fear (27, 39); higher knowledge paradoxically predicts greater fear. Khalil et al. (35) found that high knowledge combined with high fear reduces willingness to change behavior. Thus, simply imparting disease information may be ineffective or even counterproductive, leading older adults to avoid screening. Health education should therefore avoid overemphasizing irreversibility and instead pair risk information with actionable preventive strategies such as exercise and cognitive training, alongside psychological coping training, to help older adults perceive risk as manageable.

Subjective memory complaints are consistently associated with fear (20, 28), but the relationship between objective cognitive function and fear remains unclear. Experimental studies indicate that fear primarily impairs executive function rather than memory, and only under stereotype threat conditions (38, 41). This suggests that fear affects cognitive performance not through direct impairment but by activating negative age stereotypes. Older adults who worry about dementia perform worse when exposed to the implicit message that older adults have poor cognitive abilities, thereby confirming their own fears. Interventions that reduce fear may disrupt this self-fulfilling cycle and indirectly protect cognitive function.

### 4.5 Social exposure and support

The consistency of these findings is noteworthy; however, the specific construct measured (e.g., fear vs. worry) and its dimensional structure may influence how social factors are associated with dementia-related distress. A positive relationship between a history of exposure to dementia and fear has been consistently reported (30, 32, 39), but the nature and depth of such exposure may determine the direction of the effect. Brief exposure may increase awareness of the severity of the disease, while in-depth exposure may reduce fear by dispelling the sense of mystery. Most existing studies treat exposure history as a dichotomous variable, failing to distinguish between quality and intensity. Social support is negatively correlated with fear (32, 36), but the effects of instrumental, emotional, and informational support may differ; existing studies have not carefully distinguished among them.

### 4.6 Intervention evidence

It is important to consider that the two intervention studies targeted somewhat different constructs (dementia worry vs. fear of Alzheimer's disease), which may limit direct comparability of their effects. Two randomized controlled trials provide preliminary evidence of the intervention's effectiveness (29, 37). Modified group cognitive behavioral therapy demonstrated a large effect size, with particularly significant results among older adults with low educational attainment (37); however, since the sample was drawn from nursing homes, the generalizability of these findings is unclear. Although brief psychological interventions showed efficacy, the differences between groups were not statistically significant (29), which may be related to the fact that the control group received effective psychoeducational components. Intervention designs need to distinguish between specific active components and nonspecific factors. Overall, the evidence base for these interventions remains in its early stages, consisting of only two small-sample randomized controlled trials, and lacks long-term follow-up, cross-cultural validation, and cost-benefit analyses.

## 5 Limitations

This study has the following limitations: (i) the search was limited to Chinese and English databases and did not include gray literature, which may result in publication bias; (ii) the vast majority of included studies employed cross-sectional designs, making it impossible to establish causal relationships and insufficient to support causal inferences; (iii) sample selection bias was prominent, with most participants being white, female, and highly educated, and geographic distribution was uneven; (iv) significant inconsistencies in measurement tools limited cross-study comparisons; (v) no formal risk-of-bias or methodological quality assessment was conducted. This decision is consistent with the scoping review objective of mapping the breadth of evidence rather than evaluating study quality; consequently, the narrative synthesis should be interpreted as descriptive rather than evidential, and findings from the small-sample RCTs should be treated as preliminary; (vi) the two intervention studies specifically recruited participants with high baseline dementia fear (i.e., the “worried well”). This sampling strategy limits the generalizability of intervention effects to community-dwelling older adults with mild or subclinical levels of dementia fear. (vii) The search was restricted to English- and Chinese-language publications because the review team was proficient only in these two languages. This restriction may introduce language bias, and relevant studies published in other languages (e.g., Japanese, Korean, German, French) may have been missed.

## 6 Conclusions and clinical recommendations

This review highlights the current state of research on dementia fear among older adults: there is a lack of standardized measurement tools; while most studies focus on mild to moderate levels of fear, the distribution of research is uneven; and while gender and depression show the strongest associations, the causal relationships remain unclear. Future efforts should focus on developing cross-culturally standardized measurement tools, conducting longitudinal studies to distinguish between age- and generation-specific effects, designing large-sample randomized controlled trials to test the specific effects of interventions, and paying particular attention to individuals with moderate levels of anxiety. Given the close association between dementia fear and depression and insomnia, it is recommended that this be incorporated into routine mental health assessments for older adults to enable early identification of high-risk individuals and provide targeted support. As the aging population accelerates, in-depth research on this phenomenon holds urgent public health significance.

## Data Availability

The original contributions presented in the study are included in the article/Supplementary Material, further inquiries can be directed to the corresponding author.
